# Human Serum Supplementation Promotes Streptococcus mitis Growth and Induces Specific Transcriptomic Responses

**DOI:** 10.1128/spectrum.05129-22

**Published:** 2023-04-04

**Authors:** Yahan Wei, Camille I. Sturges, Kelli L. Palmer

**Affiliations:** a Department of Biological Sciences, The University of Texas at Dallas, Richardson, Texas, USA; The Ohio State University Division of Biosciences

**Keywords:** *Streptococcus mitis*, serum adaptation, RNA-seq, growth promotion

## Abstract

Streptococcus mitis is a normal member of the human oral microbiota and a leading opportunistic pathogen causing infective endocarditis (IE). Despite the complex interactions between S. mitis and the human host, understanding of S. mitis physiology and its mechanisms of adaptation to host-associated environments is inadequate, especially compared with other IE bacterial pathogens. This study reports the growth-promoting effects of human serum on S. mitis and other pathogenic streptococci, including S. oralis, S. pneumoniae, and S. agalactiae. Using transcriptomic analyses, we identified that, with the addition of human serum, S. mitis downregulates uptake systems for metal ions and sugars, fatty acid biosynthetic genes, and genes involved in stress response and other processes related with growth and replication. S. mitis upregulates uptake systems for amino acids and short peptides in response to human serum. Zinc availability and environmental signals sensed by the induced short peptide binding proteins were not sufficient to confer the growth-promoting effects. More investigation is required to establish the mechanism for growth promotion. Overall, our study contributes to the fundamental understanding of S. mitis physiology under host-associated conditions.

**IMPORTANCE** S. mitis is exposed to human serum components during commensalism in the human mouth and bloodstream pathogenesis. However, the physiological effects of serum components on this bacterium remain unclear. Using transcriptomic analyses, S. mitis biological processes that respond to the presence of human serum were revealed, improving the fundamental understanding of S. mitis physiology in human host conditions.

## INTRODUCTION

Streptococcus mitis is a member of the mitis group streptococci (MGS) and a pioneer human oral colonizer (1, 2). Through invasive dental procedures (e.g., tooth extraction and scaling) as well as daily dental hygiene activities (e.g., toothbrushing and flossing), S. mitis gains access to the bloodstream and causes transient bacteremia, imposing a health risk to people with compromised immunity (3). Indeed, S. mitis is one of the leading causes of bacteremia and infective endocarditis (IE), a disease that presents a mortality rate of ~18% to 40% (4–6). Conversely, S. mitis also has beneficial functions, as it can produce antimicrobials that inhibit the growth of human gingivitis pathogens (7, 8), and its lysate can activate the aryl hydrocarbon receptor (AhR) of oral epithelial cells, leading to wound healing (9). Moreover, decreased S. mitis in the oral microbiota is a biomarker for pancreatic cancer (10). However, the specific molecular mechanisms underlying interactions between S. mitis and the human host remain largely unknown. Furthermore, S. mitis physiology, compared to other bacteremia and IE bacterial pathogens (e.g., Staphylococcus aureus and enterococci), is poorly understood.

The current knowledge of S. mitis physiology as a human commensal and opportunistic pathogen is largely adapted from studies in another MGS, S. pneumoniae (11). S. pneumoniae is a significant human pathogen that causes ~1 million annual deaths of children <5 years old worldwide and shares > 99% 16S rRNA sequence identity with S. mitis (12–14). Many basic physiological processes and several cell surface components are conserved between S. mitis and S. pneumoniae (15–17). For example, S. mitis encodes 67 to 82% of known pneumococcal virulence factors (18). Intranasal immunization with S. mitis provides protection against S. pneumoniae (19), suggesting similarities in both cell surface structures and interactions with host immunity between these bacteria. However, the whole chromosomal DNA identity between S. mitis and S. pneumoniae is <60% (14). Genes unique to S. mitis are involved in many processes, including oligopeptide binding, amino acid metabolism, gene regulation, and virulence (17). Such genetic differences possibly contribute to the different niches these bacteria colonize in the human host and the diseases caused by each. A comprehensive understanding of S. mitis physiology, particularly in conditions that mimic human host environments encountered by this bacterium during colonization and infection, is fundamental to an understanding of its interactions with the human host.

Transcriptomic analyses provide a thorough picture of the physiological status of an organism and have been widely used in bacterial studies (20) yet are rarely applied to S. mitis. Thus far, only a few transcriptomic studies have been conducted in S. mitis, each designed to resolve (i) responses to competence stimulating peptides (CSPs) (21), (ii) interspecies communication with S. pneumoniae via secreted short peptides (22), and (iii) genetic differences against other major MGS species cultured in rich, undefined laboratory medium (23).

Here, we assessed the growth and transcriptional responses of S. mitis to human serum. When colonizing the gingival pocket in the human oral cavity, S. mitis is exposed to gingival crevicular fluid, which contains serum transudate (24). When colonizing the bloodstream, S. mitis is also constantly exposed to serum components. Thus, adaptation to human serum components is likely critical for this bacterium to colonize these environments. In a previous study, we observed that 5% human serum promoted the growth of a model S. mitis strain (25). In this study, we observed growth-promoting effects of human serum on several streptococcal species (including S. mitis, S. oralis, S. pneumoniae, and S. agalactiae) but not bacteria from other genera. Transcriptomic analyses were performed to reveal the responses of S. mitis to the addition of human serum, whose presence alleviates stresses and provides key nutrients, providing information to understand S. mitis adaptation to host-associated environments.

## RESULTS AND DISCUSSION

### Addition of human serum promotes the growth of some streptococci.

**FIG 1:**
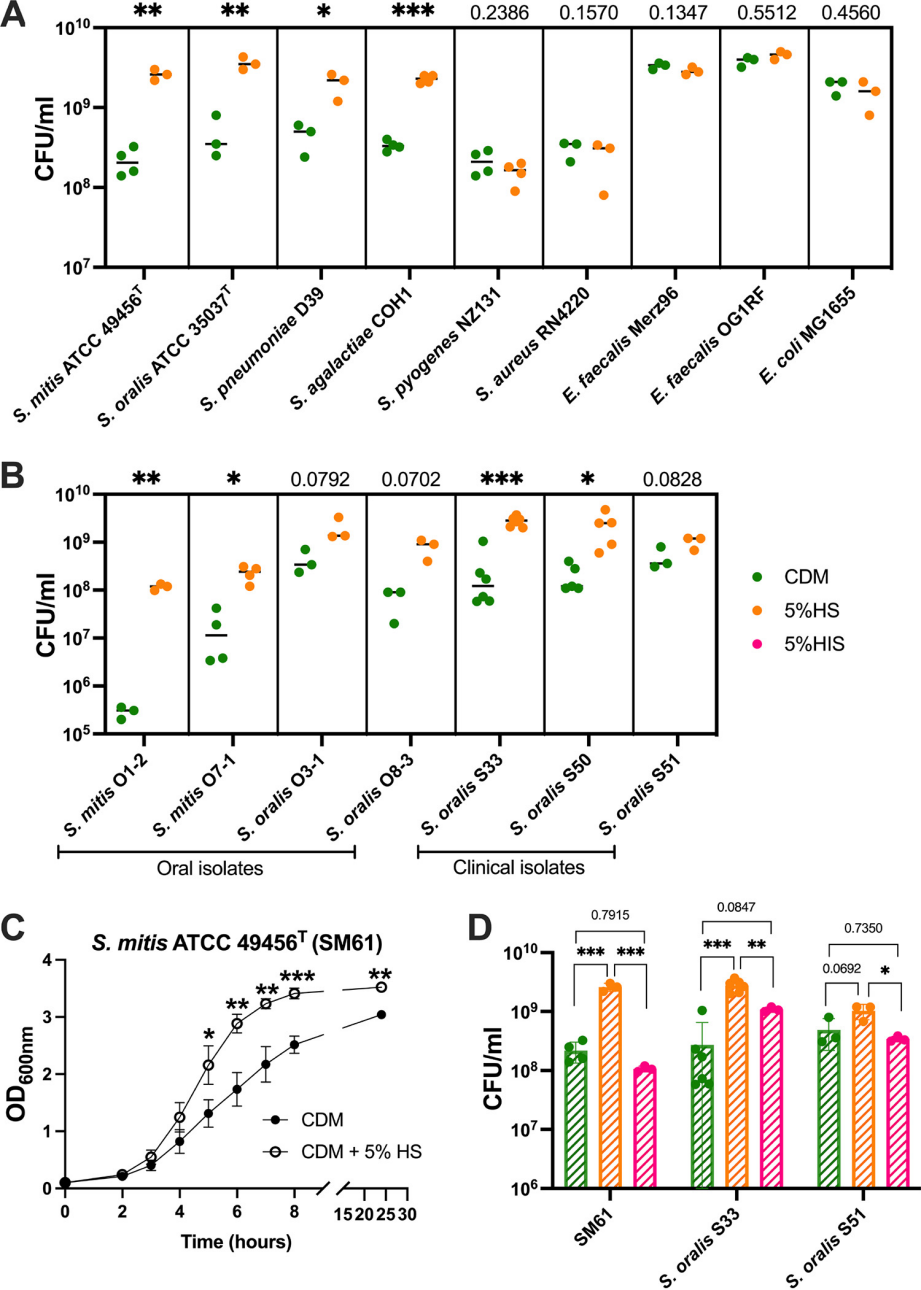
Serum supplement promotes the growth of some streptococci. (A) Cell concentrations (CFU/mL) of the indicated bacteria grown to stationary phase in either chemically defined medium (CDM; green dots) or CDM with 5% (vol/vol) supplement of complete human serum (HS; orange). (B) CFU/mL of the indicated MGS isolates grown to stationary phase in either CDM (green dots) or HS (orange dots). (C) Hourly growth curve of S. mitis ATCC 49456^T^ (SM61) grown in either CDM or CDM with 5% (vol/vol) supplement of HS. (D) Stationary-phase cell densities (CFU/mL) of SM61, S. oralis S33, and S. oralis S51 grown in CDM (green), HS (orange), or CDM with 5% (vol/vol) supplement of heat-inactivated human serum (HIS; pink). All bacterial cultures were generated from dilution of overnight bacterial cultures in CDM to an OD_600nm_ value of 0.1 followed by incubation. At least three biologically independent replicates were obtained for each tested condition. For panels A to C, statistical analyses were performed with two-tailed *t* test, with an *F* test conducted to verify whether compared groups have equal variances. Significant difference was determined by a *P* value of < 0.05. For D, one-way ANOVA was used for statistical analyses, followed with Tukey-Kramer test for multiple comparisons. Significant difference was determined by an adjusted *P* value of <0.05. Nonsignificant *P* values are labeled within graph; *, 0.01 < *P* < 0.05; **, 0.001 < *P* < 0.01; ***, *P* < 0.001.

To test how human serum addition affects bacterial growth, selected bacterial species were grown in either chemically defined medium (CDM) or CDM supplemented with complete human serum to a final concentration of 5% (vol/vol). Significant increases in cell yield as measured by CFU were observed for S. mitis, S. oralis, S. pneumoniae, and S. agalactiae cultured with human serum. This was not observed for S. pyogenes and bacteria from other genera (S. aureus, Enterococcus faecalis, and Escherichia coli) (Fig. 1A). Additional S. mitis and S. oralis strains were also tested. These strains were reported recently (26) and were isolated from the oral cavities of volunteers (Table 1, oral isolates) or from the bloodstream of hospitalized patients (Table 1, blood isolates). Human serum significantly promoted the growth of some, but not all, of the isolates (Fig. 1B).

**TABLE 1:** Strains and plasmids used in this research

Name	Description	Reference
Bacterial strains		
Escherichia coli K-12 MG1655	Model E. coli strain	77
Staphylococcus aureus RN4220	Laboratory strain of S. aureus, a mutant strain derived from clinical isolate NCTC8325	78, 79
Enterococcus faecalis		
OG1RF	Rifampin- and fusidic acid-resistant derivative of a human oral cavity isolate	80
Merz96	Vancomycin resistant strain isolated from human blood	81
Streptococcus		
S. mitis ATCC 49456^T^ (SM61)	Type strain of S. mitis	ATCC
S. mitis *ΔaliB*	SM61 with coding region of SM12261_RS06745 replaced with *ermB*	This study
S. mitis O1-2	Oral isolate obtained from healthy individual	26
S. mitis O7-1	Oral isolate obtained from healthy individual	26
S. oralis ATCC 35037^T^	Type strain of S. oralis	ATCC
S. oralis O3-1	Oral isolate obtained from healthy individual	26
S. oralis O8-3	Oral isolate obtained from healthy individual	26
S. oralis S33	Strain isolated from clinical blood sample	26
S. oralis S50	Strain isolated from clinical blood sample	26
S. oralis S51	Strain isolated from clinical blood sample	26
S. pneumoniae D39	Clinically isolated strain, serotype 2	29
S. agalactiae COH1	Clinically isolated strain, type III capsular polysaccharide	82
S. pyogenes NZ131	Clinically isolated strain, serotype M49	ATCC
Plasmids		
pMSP3535	Plasmid used to obtain *ermB* gene	83

**FIG 2:**
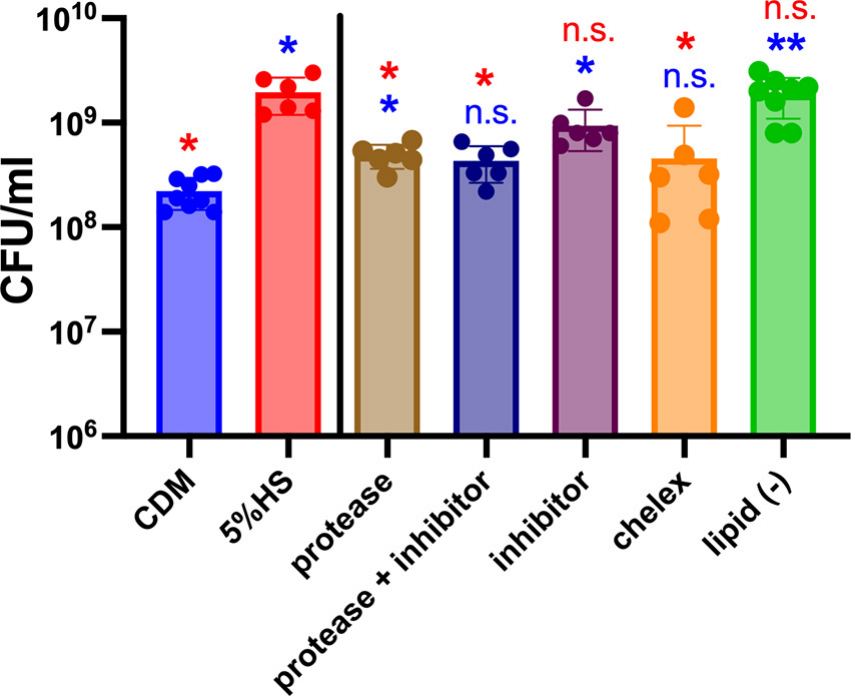
Effects of serum proteins, metal ions, and lipids in promoting growth of SM61. Stationary-phase cell densities (CFU/mL) of SM61 grown in chemically defined medium (CDM) or CDM supplemented with the indicated human serum to a final concentration of 5% (vol/vol). See Materials and Methods for how serum was pretreated prior to bacterial inoculation. Bacterial cultures were generated from dilution of overnight bacterial cultures in CDM with the indicated addition to an OD_600nm_ value of 0.1 followed by incubation. For each condition, at least six biologically independent replicates were obtained. Statistical analysis was performed with one-way ANOVA, followed with Dunnett’s test with either the CDM (significant difference indicated by blue *) or 5%HS (significant difference indicated by red *) as the control. Significance was determined by an adjusted *P* value of <0.05. *, 0.01 < *P* < 0.05; **, 0.001 < *P* < 0.01; n.s., nonsignificant (*P* ≥ 0.05).

For S. mitis ATCC 49456^T^ (SM61), the addition of human serum resulted in a 10-fold average increase of cell yield at the stationary phase (Fig. 1A) and significantly decreased generation time from 46.8 (±1.6) min in CDM to 39.1 (±0.8) min in serum-supplemented CDM with a *t* test *P* value of 0.002 (Fig. 1C). Interestingly, when supplemented with heat-inactivated serum, growth of SM61 and the clinical isolate S. oralis S33 and S51 decreased significantly compared to those grown with untreated serum (Fig. 1D), although S. oralis S51 had no significant growth promotion with the addition of untreated serum (Fig. 1D). Heat inactivation is designed to inactivate complement and will also result in aggregation of immunoglobulins (27). To verify whether serum proteins (including complement and immunoglobulins) are responsible for such growth-promoting effects, serum was digested with protease. After the treatment, protease activity was inhibited via the addition of a protease inhibitor. As shown in Fig. 2, the presence of protease in serum does result in a significant decrease in growth promotion, but it is with the combination of the inhibitor that the promoting effect is completely abolished, even though the inhibitor by itself in serum does not affect bacterial growth. These results confirmed the growth-promoting effects of serum proteins and indicated that other factors may also contribute to promoting bacterial growth.

Overall, these data suggest that temperature-sensitive component(s) is/are involved in the growth-promoting effects of human serum and that the effects of human serum on streptococcal growth are species and strain dependent.

### Transcriptomic analysis of the biological processes involved in growth promotion with serum addition.

**FIG 3:**
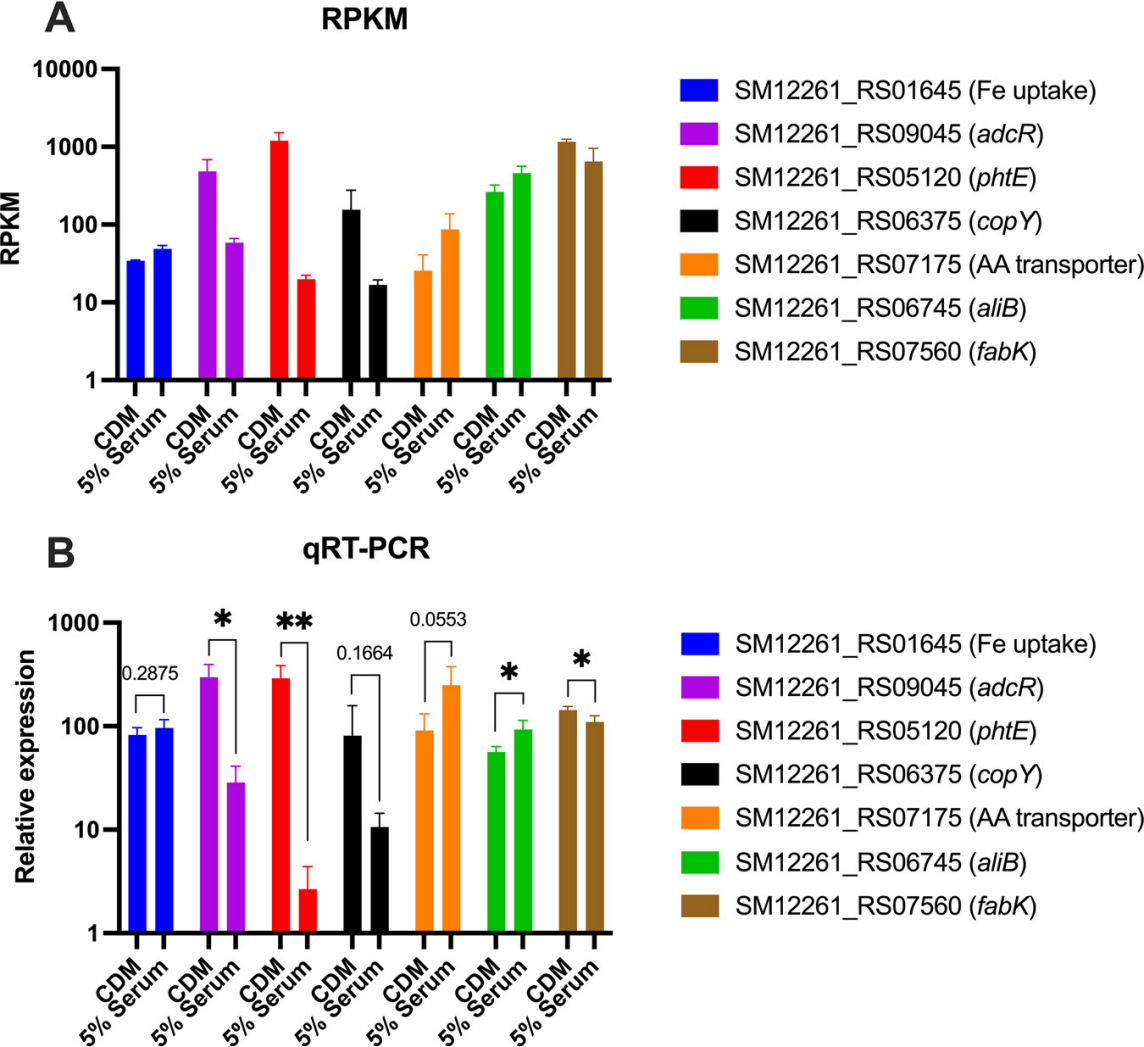
Relative expression levels of selected SM61 genes detected through RNA sequencing (A) and qRT-PCR (B). Except SM12261_RS01645 (encodes an iron utilization gene), all other genes are differentially expressed in RNA sequencing analyses. (A) RPKMs of each selected gene. (B) transcript levels of each selected gene were normalized to that of 16S rRNA from the same sample with the ΔΔ*C_T_* method. For each tested condition, three biologically independent samples were obtained. Statistical analyses were performed with two-tailed *t* test, with *F* test conducted to verify whether compared groups have equal variances or not. Significant difference was determined by a *P* value of <0.05. Nonsignificant *P* values are labeled within graph; *, 0.01 < *P* < 0.05; **, 0.001 < *P* < 0.01.

To analyze the transcriptional responses of SM61to human serum addition, RNA sequencing was performed. For these experiments, S. mitis cells in the exponential phase (~0.3 optical density at 600 nm [OD_600nm_]) exposed to 5% human serum for 30 min were compared with control, unexposed cultures. This approach was taken to minimize the confounding effects of different growth rates/yields between the two conditions on transcriptional analysis and to focus on the initial responses of S. mitis to human serum. Expression values were represented by reads per kilobase of transcript per million mapped reads (RPKM). Genes differentially regulated in response to serum were determined by ≥1.5-fold absolute changes at the transcript level, a corresponding false discovery rate (FDR) *P* value of <0.01, and a maximum RPKM of >10. By these parameters, a total of 77 downregulated genes (Table 2) and 19 upregulated genes (Table 3) were obtained, which, according to previous genomic annotations (17, 28), are all shared between S. mitis and S. pneumoniae. According to the annotated S. mitis locus structures and the transcription start sites of their homologs in S. pneumoniae (29), these down- and upregulated genes belong to approximately 48 and 13 operons, respectively (Table 2 and 3). Transcriptional changes for a subset of genes were confirmed via quantitative reverse transcriptase PCR (qRT-PCR) (Fig. 3).

**TABLE 2:** Genes downregulated upon the addition of human serum^*a*^

Operon	Locus tag	Gene name	Fold change	Protein product
Metal ion utilization systems				
1	SM12261_RS05120	*phtE*	−61.49	Pneumococcal-type histidine triad protein
2	SM12261_RS05125	*phtD*	−57.29	Pneumococcal-type histidine triad protein
	SM12261_RS05130	*adcAII*	−45.44	
3	SM12261_RS06365	*copA*	−9.07	Heavy metal translocating P-type ATPase
	SM12261_RS06370	*cupA*	−10.24	ATPase
	SM12261_RS06375	*copY*	−9.51	CopY/TcrY family copper transport repressor
4	SM12261_RS09030	*adcA*	−8.85	
	SM12261_RS09035		−9.05	Metal ABC transporter permease
	SM12261_RS09040		−8.55	Metal ABC transporter ATP-binding protein
	SM12261_RS09045	*adcR*	−8.47	MarR family transcriptional regulator (AdcR)
5	SM12261_RS07145		−1.70	Matrixin family metalloprotease
Fatty acid biosynthetic genes				
6	SM12261_RS03785	*bioY*	−2.30	Biotin (vitamin H) transporter BioY
7	SM12261_RS04060	*plsY*	−1.65	Acyltransferase
8	SM12261_RS06305	*fakB3*	−2.07	DegV family protein
9	SM12261_RS07520	*accA*	−19.82	Acetyl-CoA carboxylase carboxyl transferase subunit alpha
	SM12261_RS07525	*accD*	−16.70	Acetyl-CoA carboxylase carboxyltransferase subunit beta
	SM12261_RS07530	*accC*	−12.60	
	SM12261_RS07535	*fabZ*	−9.88	
	SM12261_RS07540	*accB*	−7.48	Acetyl-CoA carboxylase biotin carboxyl carrier protein
	SM12261_RS07545	*fabF*	−5.42	
	SM12261_RS07550	*fabG*	−3.64	
	SM12261_RS07555	*fabD*	−2.65	
	SM12261_RS07560	*fabK*	−1.83	
	SM12261_RS07565	*acpP*	−2.55	Acyl carrier protein
	SM12261_RS07570	*fabH*	−2.06	Ketoacyl-ACP synthase III
	SM12261_RS07575	*fabT*	−1.89	MarR family transcriptional regulator (FabT)
CiaRH-regulated genes				
10	SM12261_RS03850	*ciaR*	−1.99	Response regulator transcription factor
	SM12261_RS03855	*ciaH*	−1.79	HAMP domain-containing histidine kinase
11	SM12261_RS04920	*pspC*	−3.40	PspC domain-containing protein
12	SM12261_RS08965		−2.16	Bacteriocin immunity protein
	SM12261_RS08970		−1.82	CPBP family intramembrane metalloprotease
	SM12261_RS09105	*dltX*	−1.90	Teichoic acid d-Ala incorporation-associated protein
14	SM12261_RS09395	*htrA*	−2.06	Serine protease (htrA)
	SM12261_RS09400	*parB*	−1.82	ParB/RepB/Spo0J family partition protein
Other genes				
15	SM12261_RS00235		−1.78	*N*-acetylmuramoyl-l-alanine amidase family protein
16	SM12261_RS00240		−2.07	*N*-acetylmuramoyl-l-alanine amidase family protein
17	SM12261_RS00245		−1.67	GHKL domain-containing protein
18	SM12261_RS00360	*agaB*	−1.83	PTS system mannose/fructose/*N*-acetylgalactosamine-transporter subunit IIB
	SM12261_RS00365	*manY*	−1.75	PTS mannose/fructose/sorbose/*N*-acetylgalactosamine transporter subunit IIC
	SM12261_RS00370	*manZ*	−2.12	PTS mannose/fructose/sorbose transporter family subunit IID
	SM12261_RS00375	*manX*	−2.62	PTS sugar transporter subunit IIA
	SM12261_RS00380		−2.44	SIS domain-containing protein
19	SM12261_RS00390	*galM*	−2.67	Galactose mutarotase (first step of Leloir pathway), lactose catabolism
20	SM12261_RS00605		−1.87	Hypothetical protein
21	SM12261_RS00955		−2.51	Prepilin-type N-terminal cleavage/methylation domain-containing protein (type II secretion)
22	SM12261_RS01340		−2.85	Hypothetical protein
23	SM12261_RS01350	*rpmH*	−1.78	50S ribosomal protein L34
24	SM12261_RS01545		−2.68	Helix-turn-helix transcriptional regulator
25	SM12261_RS01820		−2.37	CPBP family intramembrane metalloprotease; ydiL/ abi family protein
26	SM12261_RS01950		−2.06	*N*-acetylmuramoyl-l-alanine amidase family protein
27	SM12261_RS02345		−1.63	CsbD family protein
28	SM12261_RS02365		−1.60	GlsB/YeaQ/YmgE family stress response membrane protein
29	SM12261_RS02535	*trxA*	−1.66	Thioredoxin (sulfur utilization)
30	SM12261_RS02950	*thrS*	−1.76	Threonine-tRNA ligase
31	SM12261_RS03245	*rpsU*	−2.72	30S ribosomal protein S21
32	SM12261_RS03795		−1.62	HlyD family efflux transporter periplasmic adaptor subunit (pseudogene)
	SM12261_RS03800		−1.60	ABC transporter ATP-binding protein
	SM12261_RS03805		−1.70	FtsX-like permease family protein
33	SM12261_RS03935		−2.92	DUF1797 family protein
34	SM12261_RS04005		−1.66	30S ribosomal protein S20
35	SM12261_RS04335		−2.09	Lactoylglutathione lyase
36	SM12261_RS04915		−1.72	ABC transporter ATP-binding protein
37	SM12261_RS05070		−2.08	4-Oxalocrotonate tautomerase
38	SM12261_RS05140	*sdbB*	−1.82	TlpA family protein disulfide reductase
	SM12261_RS05145	*ccdA2_1*	−1.68	Cytochrome *c* biogenesis protein CcdA
39	SM12261_RS05150		−2.26	HU family DNA-binding protein
40	SM12261_RS05235		−1.93	NADP-dependent glyceraldehyde-3-phosphate dehydrogenase
41	SM12261_RS05915		−2.09	LPXTG cell wall anchor domain-containing protein
	SM12261_RS05920		−1.79	Hypothetical protein (partial)
42	SM12261_RS06760		−1.69	CsbD family protein
43	SM12261_RS06925		−2.45	DUF3953 domain-containing protein
44	SM12261_RS07665	*liaS*	−1.62	Sensor histidine kinase
	SM12261_RS07670	*liaF*	−1.59	Transporter
45	SM12261_RS08265		−2.54	50S ribosomal protein L28
46	SM12261_RS08730		−2.44	Membrane protein
47	SM12261_RS08845		−2.14	Hypothetical protein
48	SM12261_RS08925		−1.65	Glycosyltransferase

a PTS, phosphotransferase system.

**TABLE 3:** Genes upregulated upon the addition of human serum

Operon	Name	Gene	Fold change	Protein product
CiaRH-regulated gene				
1	SM12261_RS01270	*manL*	1.69	Mannose-specific IIB subunit of PTS system
Metalloenzymes				
2	SM12261_RS00935		4.15	Zinc-dependent alcohol dehydrogenase family protein
3	SM12261_RS01275	*adhP*	4.99	Alcohol dehydrogenase
4	SM12261_RS01285		1.54	NCS2 family permease (nucleobase transporter)
5	SM12261_RS06110		1.51	FAD: protein FMN transferase
	SM12261_RS06115		1.51	NAD(P)H-dependent oxidoreductase
6	SM12261_RS09120	*glpO*	1.99	Type I glycerol-3-phosphate oxidase
Amino acid transportation				
7	SM12261_RS01690	*glnA*	1.95	Type I glutamate-ammonia ligase
8	SM12261_RS05465		1.98	ABC transporter permease subunit
	SM12261_RS05470		1.80	Amino acid ABC transporter ATP-binding protein
9	SM12261_RS07175		3.32	Amino acid ABC transporter ATP-binding protein
	SM12261_RS07180		3.84	Transporter substrate-binding domain-containing protein
	SM12261_RS07185		3.32	Amino acid ABC transporter permease
	SM12261_RS07190		2.83	Amino acid ABC transporter permease
Peptide uptake				
10	SM12261_RS06745	*aliB*	1.70	Peptide ABC transporter substrate-binding protein; *amiA aliA/B* homolog
11	SM12261_RS07850		1.75	Peptide ABC transporter substrate-binding protein (AliB-like ORF2)
Hypothetical proteins				
12	SM12261_RS02965		1.85	ABC transporter ATP-binding protein
13	SM12261_RS08945		1.76	DUF4097 family beta strand repeat protein
	SM12261_RS08950		1.76	Hypothetical protein

In general, the regulated genes are involved in biological processes of (i) transportation of metal ions (zinc and copper), sugars, amino acids, and short peptides; (ii) fatty acid biosynthesis, including type II fatty acid biosynthetic (FASII) genes, an acyltransferase, and an exogenous fatty acid binding protein; (iii) putative stress sensing regulators and stress response genes; and (iv) proteins with unknown functions (Table 2 and 3).

### Downregulation of metal ion transportation.

The most downregulated genes in S. mitis SM61 supplemented with serum are *phtE*, *phtD*, and *adcII*, which are associated with Zn utilization (Table 2). Other Zn utilization genes of the *adcR* locus are also downregulated >5-fold. While zinc is present in human serum at an estimated concentration of 9 to 17 μM (30, 31), CDM does not contain added Zn. Thus, the addition of serum increases the amount of available Zn. S. mitis likely downregulates Zn uptake genes after serum addition to maintain metal ion homeostasis. However, the addition of Zn into CDM does not result in accelerated growth or an increase of the stationary-phase OD_600nm_ values of S. mitis (Fig. S1 in the supplemental material). Interestingly, the removal of divalent cations from serum by Chelex treatment abolished the growth-promoting effects of 5% serum on SM61 (Fig. 2). Therefore, although increased Zn availability alone does not explain the growth-promoting effects of human serum, divalent cations are involved in growth promotion.

In S. pneumoniae, the cellular concentration of Zn affects the expression of Zn uptake genes and those involved in Zn export (*sczD*) (32), Mn uptake (*psaABC*) (33), and Cu export (*copA*) (34) through modulating activities of their corresponding transcriptional regulators. Among these genes, homologs of the *copY*-*cupA*-*copA* operon that encodes a transcriptional repressor (CopY), Cu chaperone (CupA), and Cu exporter (CopA) are downregulated in S. mitis upon serum addition. In the presence of Zn, pneumococcal CopY binds DNA and represses transcription of its target genes (34); thus, the observed downregulation of the *copY*-*cupA*-*copA* likely results from increased Zn abundance due to serum addition. Interestingly, other genes, including *sczD* and *psaABC*, that are expected to be regulated in response to increased Zn availability were not differentially expressed in our data set (Table S1), suggesting a hierarchical regulatory relationship among these metal utilization systems in S. mitis.

### Downregulation of fatty acid biosynthetic genes.

Aside from the Zn utilization genes, genes encoding the acetyl-CoA carboxylase complex (*accADC*) are the next most downregulated genes in this data set for fold changes >10 (Table 2). These genes belong to the FASII pathway for *de novo* fatty acid biosynthesis. In S. pneumoniae, the incorporation of exogenous lipids downregulates the expression of FASII genes through the activities of the transcriptional regulator FabT (35). Human serum is a rich source of lipids (36). Evidence that streptococci utilize serum fatty acids already exists (37, 38); thus, the downregulation of the FASII genes in S. mitis with the addition of human serum is intuitive. Indeed, all FASII genes are downregulated in S. mitis after serum supplementation (Table 2).

Interestingly, downregulation of the biotin transporter gene *bioY* (SM12261_RS03785), a homolog of the exogenous fatty acid binding protein FakB3 (SM12261_RS06305), and acyltransferase gene *plsY* (SM12261_RS04060) were also observed. Biotin is a cofactor for FASII carboxylation enzymes (39). Thus, it is possible that the expression of *bioY* correlates with that of the FASII genes, through an as yet unknown regulatory mechanism in S. mitis. Both *fakB3* and *plsY* are involved in fatty acid utilization. Specifically, exogenous fatty acid is recognized and bound by a cell surface protein (FakB), phosphorylated by a fatty acid kinase (FakA), and then used for the synthesis of phosphatidic acids by acyltransferases that include PlsY (40). S. mitis encodes three copies of *fakB*, SM12261_RS06970, SM12261_RS05155, and SM12261_RS06305, corresponding to homologs of the pneumococcal fatty acid binding protein FakB1, FakB2, and FakB3. In S. pneumoniae, these FakB proteins recognize fatty acids that differ by their acyl chain length and saturation status (41). The exact functions of S. mitis FakB proteins still await further studies, as do the transcriptional regulatory mechanisms that control the expression of *fakB* genes. Interestingly, among the three *fakB* genes, only *fakB3* was differentially expressed after serum addition. Similarly, among the three known acyltransferases in S. mitis (*plsC* SM12261_RS03010, *plsY*, and *plsX* SM12261_RS00220), only *plsY* was differentially expressed after serum addition. Such differences in expression status suggest different functions of these genes and/or different regulatory systems that individually fine-tune the expression of these genes in response to environmental changes.

Many host-derived fatty acids are potent antimicrobials (42). Although streptococci are able to utilize exogenous lipid metabolites (37, 41), growth promotion by lipid addition has not been reported before. To further evaluate whether serum lipids participate in promoting bacterial growth, the stationary-phase cell densities of SM61 grown with lipid-depleted serum were measured and compared to growth in untreated serum. No significant difference was observed (Fig. 2), suggesting that serum lipids are not the major component contributing to growth promotion.

### Downregulation of stress sensing regulators and stress response genes.

Several genes with activities in transcriptional regulation and stress response are downregulated in response to human serum, suggesting that serum addition alleviates some stresses, potentially triggering growth-promoting effects. Among these regulatory systems, homologs of the two-component system (2CS), CiaRH (*ciaR*: SM12261_RS03850; *ciaH*: SM12261_RS03855), and *liaFS* (*liaF*: SM12261_RS07670; *liaS*: SM12261_RS07665), which are part of the three-component system (3CS) LiaFSR, are downregulated, as well as genes that are predicted to be regulated by these systems. LiaFSR is conserved among several Gram-positive bacteria (43–46), senses structural and compositional changes in the bacterial membrane and cell wall components, and mediates stress responses (47, 48). Thus, the downregulation of *liaFS* possibly results from altered lipid compositions caused by the addition of serum, as indicated by the downregulation of FASII genes (Table 2). Among the known operons that are directly regulated by LiaR (49), besides the *liaFS* genes, only the *pspC* homolog was differentially expressed, which is also regulated by CiaR.

CiaRH is a conserved 2CS shared among many pathogenic streptococcal species, including S. pneumoniae, S. agalactiae, and S. pyogenes (50). It mediates resistance to stresses such as oxidative stress, acid, and antibiotics (51) and controls several growth and virulence-related processes such as cell wall biosynthesis and biofilm formation (15, 49, 50, 52). In response to unknown environmental stimuli, membrane-embedded histidine kinase CiaH phosphorylates transcriptional regulator CiaR, which activates its DNA binding ability (53). Additionally, acetyl phosphate and other histidine kinases can also activate CiaR (53). In S. pneumoniae, CiaR directly regulates 18 promoters (49). Among those regulated genes, homologs to the *htrA*-*parB* operon for replication partition (SM12261_RS09395-400), bacteriocin production (SM12261_RS08965-70), cell wall d-alanine modification *dltX* (SM12261_RS09105), virulence gene *pspC* (SM12261_RS04920), and the first gene of a mannose transporter operon (SM12261_RS01270) were all differentially regulated in response to human serum (Table 2 and 3). Six of the 18 CiaR-regulated promoters in S. pneumoniae drive expression of noncoding RNAs (ncRNAs) (49); however, the profile of S. mitis ncRNAs is still unclear; thus their expression levels were not included in this analysis.

### Genes upregulated in the presence of human serum.

Nineteen genes were upregulated by S. mitis after the addition of human serum (Table 3). These genes include a sugar uptake gene putatively regulated by CiaRH, six metalloenzyme genes possibly regulated in response to changes in metal ion availability, seven amino acid transportation genes, two short peptide uptake genes, and three hypothetical genes of unknown functions.

The glutamine synthetase GlnA is involved in regulating bacterial nitrogen metabolism, including glutamine uptake (54). Additionally, the expression of glutamine transporters is coregulated by transcriptional regulators, including CodY and GlnR (54, 55), which were not differentially regulated in our data set. Bioinformatic analyses identified at least six glutamine transporters in S. pneumoniae (56). S. mitis encodes homologs of each of the six systems (Table S2), only one of which (SM12261_RS07175-90) is differentially regulated with serum addition. However, qRT-PCR did not confirm the statistical significance of the upregulated transcription of SM12261_RS07175 (Fig. 3B). Further studies are needed to clarify the detailed nitrogen metabolic processes in S. mitis.

**FIG 4:**
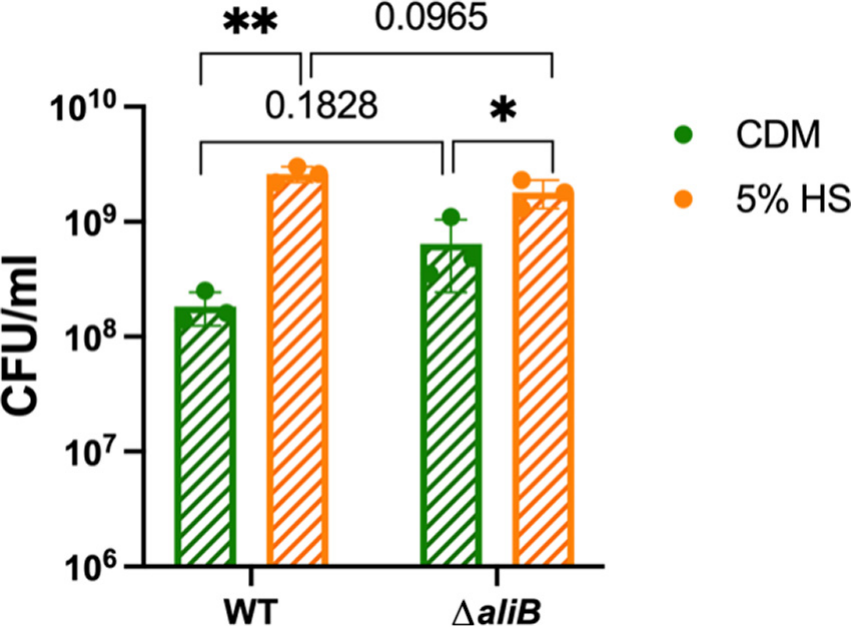
Deletion of *aliB* does not abolish the growth-promoting effects of human serum. Overnight cultures of SM61WT and *ΔaliB* were subcultured to 0.1 of OD_600nm_ with fresh CDM with and without supplement of 5% (vol/vol) human serum (HS). Cell concentrations (CFU/mL) were determined after 8 h of incubation. Three biologically independent replicates were obtained for each tested condition. Statistical analyses were performed with *t* test, with significant difference determined by a *P* value of <0.05. Nonsignificant *P* values are labeled within graph; *, 0.01 < *P* < 0.05; **, 0.001 < *P* < 0.01.

Streptococci can uptake short peptides as environmental signals and correspondingly modulate cellular processes (57). One such example is the induction of natural competence in mitis group streptococci by CSP (58). Environmental short peptides are imported through oligopeptide permeases, with different peptide-binding proteins recognizing peptides of different sequences (59). S. mitis encodes at least seven different peptide-binding proteins (Table S1), among which, SM12261_RS06745 (homolog to S. pneumoniae *aliB*) and SM12261_RS07850 (*aliB*-like ORF 2, referred to here as ORF 2) are significantly upregulated after serum addition. In S. pneumoniae, the addition of peptide ligands of AliB or ORF 2 to culture media can boost bacterial growth (60, 61). To verify whether AliB is responsible for the growth-promoting effect of human serum, a Δ*aliB* deletion mutant was generated in S. mitis. However, no significant growth difference was observed between WT and *ΔaliB* SM61 that were grown in either plain CDM or serum-supplemented CDM (Fig. 4), and the growth-promoting effect of serum was still observed for *ΔaliB* (Fig. 4). Considering that deletion of the ORF2 gene in S. pneumoniae also failed to abolish the growth-promoting effect of the corresponding peptide ligand (61), it is possible that these peptide receptors have redundant functions in ligand binding. Additionally, human serum, as a complex nutrient source, could also contain ligands for both receptors. Further studies are needed to resolve the possible roles of *aliB* and ORF 2 in mediating S. mitis serum-associated growth promotion.

### Conclusions.

In this study, we explored the effects of serum supplementation on the growth of S. mitis and a range of other bacteria that cause bloodstream infections and IE. We observed substantial growth promotion of some, but not all, species and strains tested in the presence of serum. Studies that analyze a larger strain cohort and identify conserved genetic features in strains exhibiting enhanced growth in serum (e.g., with a genome-wide association study) could reveal a mechanistic basis for this variability. It would also be useful to query multiple strains of S. pneumoniae, S. pyogenes, S. agalactiae, and other bacteria used in this study to determine whether variability is also observed. A weakness of our study is the limited number of isolates used for these species.

The mechanism(s) for the observed growth promotion was not elucidated in this study. According to the transcriptomic results, S. mitis encounters an environment with more abundant nutrient sources upon the addition of serum and particularly favors the utilization of exogenous lipids rather than *de novo* biosynthesis. Although not fully characterized, factors in serum that promote the growth of S. mitis seem to be heat labile and divalent cation dependent, and the growth promotion is possibly conferred by multiple factors, including proteins. One of the major known heat-labile proteins in serum is the complement, which has antimicrobial activities (62). Although MGS strains have been reported with different complement-binding properties (63), complement-induced growth has not been reported before. Moreover, the known virulence factor that confers resistance to complement, PspC (64), is downregulated in this data set.

Interestingly, the type I lipoteichoic acid (LTA) synthase gene *ltaS* seems related to the serum-induced growth promotion, as its deletion in SM61 results in deficient growth in 5% serum-supplemented CDM and sensitizes SM61 to 95% complete serum (25). These results further correlate the growth-promoting effects with the heat-labile killing effects of serum. It is noted that differential expression of *ltaS* is not seen in this data set, which highlights a limitation of transcriptomic analyses in providing mechanistic information beyond transcript levels. Additionally, although the existence of type I LTA, the product of LtaS, has been confirmed in S. mitis (65), neither the *ltaS* gene nor full-length type I LTA has been detected in S. pneumoniae or S. oralis (15, 66), two other closely related MGS that also demonstrated growth promotion by serum (Fig. 1A and B). Moreover, the growth of another type I LTA-producing bacteria (e.g., S. aureus [67] and E. faecalis [68]) was not promoted with serum addition. Thus, the underlying mechanism(s) for *ltaS*-involved serum-induced growth promotion is likely to be unique to S. mitis.

Previously known serum factors that can promote bacterial growth are catecholamines (including dopamine, adrenaline, etc.) and hemoglobin, which are mainly utilized by bacteria as iron sources (69–71). However, no homologs to known transporters that uptake those factors as iron sources have been identified in S. mitis, and no iron utilization genes were differentially regulated in our data set (Table S1 and Fig. 3).

Finally, it is important to note that CDM, while defined, is also chemically rich, providing amino acids, vitamins and other cofactors, and glucose, among other components. *In situ*, S. mitis would harvest many of these components from the host or synthesize them. An informative experiment moving forward could be to compare S. mitis gene expression in buffer with serum or heat-inactivated serum, which could better illuminate S. mitis metabolic processes.

## MATERIALS AND METHODS

### Bacterial strains and culture conditions.

All bacterial strains were grown at 37°C, with Streptococcus strains grown with 5% CO_2_ supplementation. Unless otherwise stated, Streptococcus strains were grown in Todd Hewitt medium (TH medium; BD Biosciences), with S. pneumoniae and S. pyogenes strains grown in TH medium supplemented with 0.5% yeast extract. E. coli were grown in Luria-Bertani medium. *Enterococcus* strains were grown in brain-heart infusion medium. CDM was made as previously described, with the addition of 0.5 mM choline (72). Where noted, human serum was added to a final concentration of 5% (vol/vol), and Zn was added to a final concentration of 850 nM, which is equivalent to the estimated Zn supplementation from 5% human serum (30, 31). Viable cell concentrations were determined by serial dilution with phosphate-buffered saline and quantification of CFU. All bacterial strains and plasmids used in this research are shown in Table 1. All primers used in this research are listed in Table 4.

**TABLE 4:** Primers used in this research^*a*^

Name	Sequence	Functions
Primers used for qRT-PCR analysis		
rRNA-F	GGACAGAGGTGACAGGTGGT	Primers targeting on 16S rRNA
rRNA-R	TCCGAACTGAGACTGGCTTT	
RT61Fe-F	TCATGGGGAAAACTCTTTGC	Primers targeting on SM12261_RS01645
RT61Fe-R	CTAGTTTGGCGGCTTCAGTC	
RT61Zn1-F	CAGTCAGGCAGCAGTTACCA	Primers targeting on SM12661_RS09045
RT61Zn1-R	GCAGTCAAAAACCGCTGAAT	
RT61Zn2-F	AGTCTTTGCGCCTATGCACT	Primers targeting on SM12661_RS05120
RT61Zn2-R	TTGCTCAGCCTGAATTCCTT	
RT61Cu-F	TGAGAAAGAGTGCCTGACGA	Primers targeting on SM12661_RS06375
RT61Cu-R	GCAATCAAATCAGCCAACAA	
RT61Q-F	AGCCATGCTATCTGGTGGAC	Primers targeting on SM12261_RS07175
RT61Q-R	TCCACCAAAACCTCTCCATC	
RT61pep-F	ACTCAGGAAGCGGTTCTCAA	Primers targeting on SM12261_RS06745
RT61pep-R	CATCCGCAAAGTTGATTCCT	
RT61fabK-F	GCTCTTGTCTCCCTTTGTGG	Primers targeting on SM12661_RS07560
RT61fabK-R	AGCTTCCATTCCTTCTGCAA	
Generation of Δ*aliB* (SM12261_RS06745) in S. mitis		
YW109	GATTATCTAGCAGCGGTTACCCAATCC	Amplify upstream fragment of *ΔaliB*
YW110	CCCTAGCGCTTTTTCATCTCCAGAACCTCCTG	
YW111	GAGATGAAAAAGCGCTAGGGACCTCTTTAGC	Amplify fragment with *ermB*
YW112	CAACTTCGCCGATCCGTAGCGGTTTTCAAAATTTG	
YW113	GCTACGGATCGGCGAAGTTGGAAGAAAAGTG	Amplify downstream fragment of *ΔaliB*
YW114	AGCATGAGTTTGCGTCGTTGG	

a F, forward; R, reverse.

### Treatments of human serum.

Complete human serum (Sigma-Aldrich; H6914) and heat-inactivated human serum (Sigma-Aldrich; H5667; treated with 56 ± 2°C for 1 h by the manufacturer) were purchased. Depletion of lipids from human serum was performed as previously described (73).

Removal of divalent cations from serum was achieved by adding 1 g of Chelex-100 resin (Bio-Rad) into 10 mL 10% (vol/vol) serum that was prepared with sterilized water. After shaking on a platform for 1 h at 4°C, Chelex-100 resin was removed with centrifugation at 200 × *g* for 5 min followed by filtration through a 0.22-μm pore-size sterile filter. Chelex-treated serum was mixed with an equal volume of 2× concentrated CDM to achieve a final concentration of 1× CDM and 5% (vol/vol) serum.

Removal of serum proteins was conducted through the addition of the serine protease proteinase K (EMD Millipore) to a final concentration of 200 μg/mL in 2 tubes of 5 mL complete human serum followed by incubation at 37°C for 3 h. Five milliliters of complete serum without the addition of protease was incubated simultaneously. To avoid heat inactivation of the serum, cOmplete EDTA-free Protease Inhibitor Cocktail (Roche; 11836170001) was used for the inhibition of protease activity. Specifically, one tablet of the inhibitor cocktail was dissolved in 1 mL of sterilized water to generate an inhibitor solution. After incubation, 500 μL of the inhibitor solution was added into 5 mL of serum with and without protease individually.

### RNA sequencing.

Single colonies of S. mitis ATCC 49456 (SM61) were grown in CDM overnight followed by dilution to an OD_600nm_ of 0.1 with fresh prewarmed medium into two replicates. When the OD_600nm_ values of the diluted cultures reached 0.3, complete human serum was added to one set of the cultures to a final concentration of 5% (vol/vol), followed by another 30 min of incubation at 37°C with 5% CO_2_. Then the cells were pelleted, resuspended into 450 μL media, and mixed with 900 μL RNAProtect (Qiagen) for stabilizing RNA. After incubation at room temperature for 10 min, cells were pelleted at 5,000 × *g* for 10 min and stored at −80°C prior to further processing.

Total RNA was extracted with the following procedure. Cell pellets were resuspended into 1 mL of ice-cold RNABee (Tel-Test, Inc.), transferred into Lysine Matrix B tubes (MP Biomedicals), followed by 6 cycles of bead-beating at 6.5 m/s for 45 s, with 5 min on ice between cycles (FastPrep-24 MP Biomedicals). Then, 250 μL chloroform was added to each of the samples, followed by ~30 s shaking by hand followed by incubation on ice for 10 min. After centrifugation at 17,000 × *g* for 15 min, the aqueous layer was removed and mixed with an equal volume of isopropanol, followed by incubation on ice for >1 h. RNA pellets were obtained via centrifugation at 17,000 × *g* for 15 min at 4°C, followed by two washes with ice-cold 70% ethanol. The RNA pellets were air dried before rehydration with RNase-free water.

Removal of DNA was conducted with Recombinant DNase I (Roche Diagnostics) per the manufacturer’s instructions, followed by cleaning with the GeneJET RNA Cleanup and Concentration Micro kit (Thermo Scientific). Lack of DNA contamination was confirmed with PCR using primers rRNA-F and rRNA-R (Table 4). RNA sequencing of samples with RNA integrity number above 9.4 was performed by the Genome Center at The University of Texas at Dallas (Richardson, TX). Specifically, rRNA was removed with RiboMinus kit (Invitrogen), and sequencing libraries were prepared with TruSeq Stranded mRNA Library kits (Illumina) and sequenced with Illumina Nextseq 500.

Analysis of the RNA sequencing results was performed with the CLC Genomics Workbench (version 20; Qiagen). The S. mitis ATCC 49456 genome sequence (NZ_CP028414.1) was used as a reference. Sequence reads that aligned to rRNA genes were removed from further analysis. RPKM values of each annotated gene were calculated and used for the analysis of differential expression at the transcript level. The validity of replicates was confirmed with principal-component analysis (Fig. S2) and scatterplot with linear regression using the RPKM values (Fig. S3), The *R*^2^ values of the comparisons between replicates under the same testing condition are all above 0.9. RNA sequencing data for all ORFs are shown in Table S1. Differentially expressed genes were determined by an absolute fold change of >1.5, a false discovery rate (FDR) *P* value of <0.01, and a maximum RPKM value of >10.

### Quantitative reverse transcriptase PCR.

Primers used for qRT-PCR were designed via the online program Primer3 with 60 ± 2°C set as the optimized annealing temperature (74). The specificity of the primers was confirmed with PCR using purified genomic DNA as the template and analysis of products by agarose gel electrophoresis. cDNA was generated from the total RNA samples described above using SuperScript3 (ThermoFisher) per the manufacturer’s instructions and using 1 μg total RNA as the template, followed by cleaning with the QIAquick PCR purification kit (Qiagen). cDNA concentration was measured by Nanodrop (Thermo Scientific) and diluted to 100 ng/μL for use in qRT-PCR. qRT-PCRs were prepared with AzuraQuant Green Fast qPCR Mix LoRox (Azura Genomics) per the manufacturer’s instructions, using 1 μL cDNA per reaction for the detection of 16S rRNA and 5 μL cDNA per reaction for the detection of target genes. qRT-PCRs were performed on the MyGo Pro machine (Azura Genomics) and analyzed with the MyGo Pro PCR Software (v3.2, Azura Genomics). Relative transcript levels of the target genes were calculated with the ΔΔ*C_T_* method with normalization to the Ct numbers of 16S rRNA. For each sample, at least three biological independent replicates were obtained. Statistical analysis was analyzed with two-tailed *t* test, and significant differences were determined by a *P* value of <0.05.

### Gene ortholog identification.

Due to the close evolutionary relationships and genomic similarity between S. mitis and S. pneumoniae, as they share >900 core genes (17, 75), previous studies of S. pneumoniae regulatory systems were used as the main reference for the interpretation of the putative regulatory relationships among the differentially expressed genes. Specifically, gene orthologs were identified by using the BLASTp function against the NCBI database (76). Proteins of interest from S. pneumoniae were used as input to query the nonredundant protein database of S. mitis ATCC 49456^T^ (taxid: 246201). Orthologs were determined by the lowest E value (<10^−88^) of each inquiry with a query coverage of >94%.

### Mutant generation.

S. mitis mutants were generated as described before (25). Specifically, overlapping PCR was used to sequentially join a 2-kb fragment from upstream of the target gene, a 1-kb fragment containing *ermB* in the reverse orientation, and a 2-kb fragment from downstream of the target gene. The purified 5-kb fragment was transformed into S. mitis competent cells as previously described (25). Mutant candidates were selected on media with 20 μg/mL erythromycin and confirmed with Sanger sequencing (MGH CCIB DNA Core, Cambridge, MA).

### Data availability.

Raw sequencing data were submitted to the NCBI database with BioProject ID of PRJNA784985 and BioSample accessions of SAMN23523627-32.
