# Supplementary material for: Human Serum Supplementation Promotes Streptococcus mitis Growth and Induces Specific Transcriptomic Responses
Source: Microbiol Spectr. 2023 Apr 4;11(3):e05129-22. doi: 10.1128/spectrum.05129-22 (PMC10269507; doi:10.1128/spectrum.05129-22)
Supplement: Supplemental file 1 — Supplemental material. Download spectrum.05129-22-s0001.pdf, PDF file, 0.7 MB [file spectrum.05129-22-s0001.pdf]

Supplementary materials

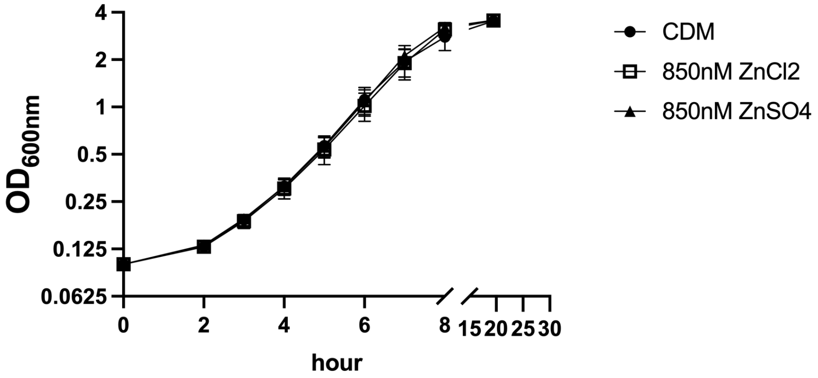

Fig S1: Growth curve of *S. mitis* ATCC 49456[T] (SM61) grown in either chemically defined medium (CDM) or CDM with 850 nM $ZnCl_2$ or $ZnSO_4$. Three biologically independent replicates were obtained for each tested condition.

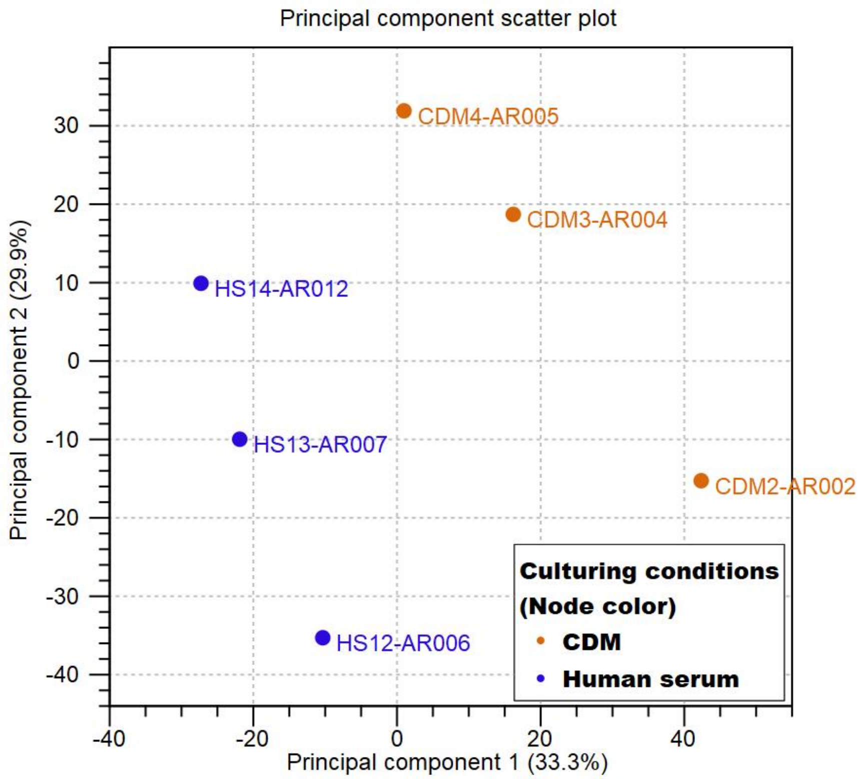

Fig S2: Plot of the principal component analysis of the RNA sequencing samples. Samples obtained from plain chemically defined medium (CDM) are represented by orange dots; while samples obtained from serum supplemented cultures are represented by blue dots. Plot figure is generated through the PCA function of the CLC Genomic Workbench.

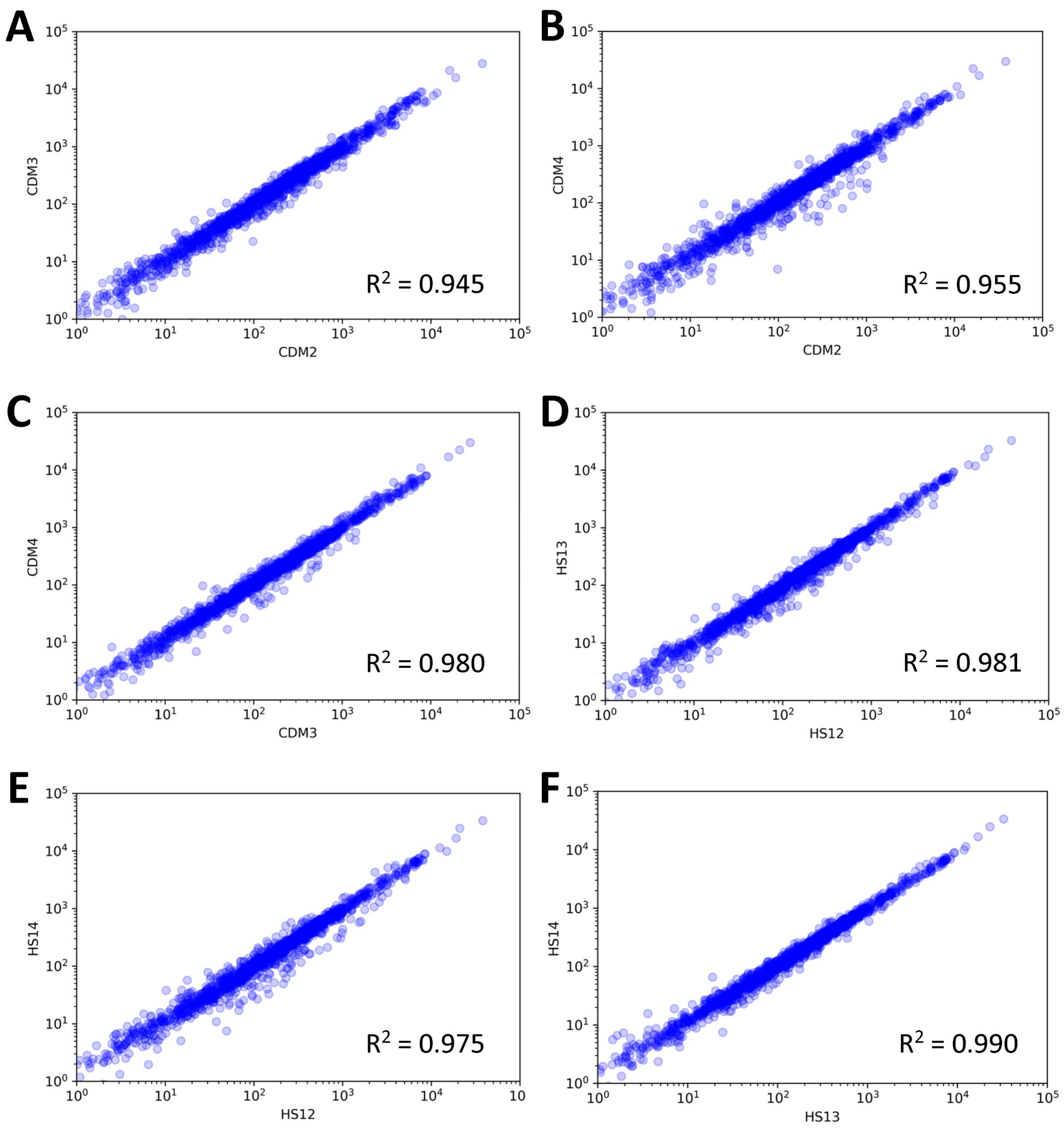

Fig S3: Replicate validation of the RNA sequencing samples via scatter plot analyses. Axis labels indicate the samples in comparison. “CDM” stands for samples grown in chemically defined medium. “HS” stands for samples grown in medium supplemented with human serum to a final concentration of 5% (v/v). R^2-values are labeled within each plot.
